# Effects of High-Intensity Interval Training and Isoinertial Training on Leg Extensors Muscle Function, Structure, and Intermuscular Adipose Tissue in Older Adults

**DOI:** 10.3389/fphys.2019.01260

**Published:** 2019-10-09

**Authors:** Paolo Bruseghini, Carlo Capelli, Elisa Calabria, Andrea P. Rossi, Enrico Tam

**Affiliations:** ^1^Department of Molecular and Translational Medicine, University of Brescia, Brescia, Italy; ^2^Department of Physical Performance, Norwegian School of Sport Sciences, Oslo, Norway; ^3^Department of Neurological and Movement Sciences, School of Sport and Exercise Sciences, University of Verona, Verona, Italy; ^4^Section of Geriatrics, Department of Medicine, University of Verona, Verona, Italy

**Keywords:** aging, high-intensity interval training, isoinertial resistance training, muscle volume, muscle architecture, muscle activation, intermuscular adipose tissue

## Abstract

We compared the effects of aerobic high-intensity training (*HIT*) and isoinertial resistance training (*IRT*) on the strength, mass, architecture, intermuscular adipose tissue (*IMAT*) quality, and neuromuscular activation of the quadriceps in elderly subjects. Twelve healthy men (69.3 ± 4.2 years; 77.8 ± 10.4 kg; 1.72 ± 0.05 m) were exposed to 8 weeks of *HIT* (7 × 2-min cycling repetitions at 90% of $\dot{V}$O_2__peak_, 3 times/week) and, after 4 months (detraining), to *IRT* (4 × 7 maximal concentric–eccentric knee extensions, 3 times/week). Before and after trainings, we measured knee extension isometric (*T*_MVC_) and dynamic (*T*_C_) maximal concentric torque, anatomical cross-sectional area (*ACSA*) at 25, 50, and 75% of femur length, quadriceps volume (*Vol*), *IMAT*, pennation angle (θ*_p_*) of the fibers from the *vastus lateralis*, and voluntary activation (*%Act*). *T*_MVC_ and *T*_C_ were significantly larger only after *IRT* (*P* = 0.008); *IRT* was able to elicit a greater increase of *ACSA* than *HIT*; *Vol* increases similarly and significantly after *HIT* and *IRT* (*P* = 0.003–0.001); *IMAT* at 50% of femur length decreased after both *HIT* and *IRT* (*P* = 0.001–0.003); physiological cross-sectional area (*PCSA*) was larger after *IRT* than before (*P* = 0.025); specific torque did not change throughout the study (45.5 N cm^–2^ ± 12.0); %*Act* of the quadriceps was significantly affected only by *IRT* (*P* = 0.011). Both *HIT* and *IRT* are able to elicit beneficial modifications of muscular mass, architecture, and quality (reducing *IMAT*) in elderly subjects in connection with an amelioration of strength. *HIT* and *IRT* caused a homogeneous increase of *ACSA* and of *Vol* of the quadriceps. *PCSA* increases, but specific strength per unit of *PCSA* did not change. The increases of functional torque seemed to be attributed to a parallel increase of %*Act* and muscle hypertrophy only after *IRT*. Data suggest that *IMAT* may be a prominent indicator to track metabolic-dependent activity and skeletal muscle quality.

## Introduction

In elderly people, the loss of muscle mass and strength has a negative impact on their daily life autonomy, balance, and gait (Narici and Maffulli, 2010). Sarcopenia (the progressive loss of muscle mass and strength with age), however, has also functional and metabolic consequences: the progressive decrease of lean body mass is mirrored by the decay of resting metabolic rate (Narici and Maffulli, 2010), and it also implies a decrease of daily physical activities and of total energy expenditure (Vaughan et al., 1991; Goran and Poehlman, 1992). Both these factors predispose elderly people to accumulate visceral and total body fat (Kohrt and Holloszy, 1995) and to develop poor insulin sensitivity and increased post-prandial hyperglycemia.

Furthermore, also, the quality of the muscles decays in the elderly because of the substantial increase of the so-called intermuscular adipose tissue (*IMAT*) (Marcus et al., 2010; Zoico et al., 2010). Unlike subcutaneous adipose tissue, *IMAT*, which might be viewed as a peripheral ectopic fat depot, surrounds and infiltrates muscle groups with which it shares a direct vascular connection. This anatomic relationship is analogous to that of visceral liver and abdominal fat, suggesting that *IMAT* might have a functional negative influence on skeletal muscle metabolism analogous to that of visceral adipose tissue on liver metabolism (Durheim et al., 2008). In spite of its role, thigh *IMAT* has not been widely studied.

It is commonly observed that long-term heavy strength training increases anatomical cross-sectional area (*ACSA*) and muscle volume (*Vol*) mainly because of preferential Type-II fiber hypertrophy (Tesch, 1988). Yet, there seems to be a selective growth within the muscles involved in training (Housh et al., 1992; Narici et al., 1996) that may depend on the magnitude of their activation due to the load imposed by the mechanic gain of each single muscle belly (Folland and Williams, 2007). However, the data are not completely consistent, since it has not been clarified whether selective hypertrophy is more pronounced, for instance, in the region of the maximal *ACSA* of the quadriceps (Häkkinen et al., 2000) or in the more distal and proximal regions of the muscles (Narici et al., 1996), also as a consequence of different training modalities and, therefore, regional loads. Differential adaptations are likely to alter the moment of inertia of the thigh and the functional consequences (Earp et al., 2015). The architectural changes must be properly taken into account when we aim to estimate the possible changes of specific tension elicited by training, since force must be normalized by the so-called physiological cross-sectional area (*PCSA*) and not by the *ACSA*. In this regard, it is not clear whether specific torque (i.e., the torque per unit of *PCSA*) may change following resistance training. In addition, any decrease in *IMAT* content brought about by training would, however, modify the specific strength calculated as the ratio between torque and *ACSA*.

In the elderly, the progressive loss of muscle mass and strength (sarcopenia) is associated with metabolic alterations. Furthermore, the peripheral changes that compromise gas exchanges contribute to the progressive decrease in the maximum cardiovascular oxygen transport observed in aging. It is therefore ecological to propose strength training. It is useful to counteract the loss of muscle mass by promoting or enhancing the beneficial adaptations induced also by aerobic training on the main risk factors leading to metabolic syndrome. Even though traditional calisthenic workout (whole-body exercises, 3 sets of 10–15 reps, 3 days/week) has been described to improve health-related parameters as biomarkers (Tomeleri et al., 2016) and body fat (Cunha et al., 2017), resistance training is mainly prescribed in elderly people to promote the increase of muscle strength and mass (Hurley et al., 1994; Hunter et al., 2004). Since resistance training is highly effective when concentric and eccentric contractions are repeatedly applied (Berg and Tesch, 1994; Franchi et al., 2017), the so-called isoinertial resistance training (*IRT*) performed with the YoYo^®^ ergometer, which implements the flywheel principle and is able to generate resistance force during both the lengthening and shortening actions of the contraction thanks to eccentric overload (Tesch et al., 2017), seems to be a promising and effective way to induce a fast increase of muscle mass and strength also in elderly people (Maroto-Izquierdo et al., 2017).

The improvement in muscle strength following resistance training is also the result of beneficial neuromuscular adaptions. The main neurological adaptations, which seem to establish already in the early phases of training, include a decrease of the activation of the antagonist muscles and enhanced agonist muscle activation due to increased motor recruitment, firing frequency, and synchronization of the motor units (Gabriel et al., 2006; Folland and Williams, 2007). By using the interpolated twitch technique to measure the level of muscular activation during maximal, voluntary isometric contraction (Shield and Zhou, 2004), several studies have indeed shown that increased muscle activation follows strength training (Shield and Zhou, 2004). Even if a high-intensity interval training (*HIT*) program induced beneficial muscular neural adaptations in young adults (Vera-Ibañez et al., 2017), no evidence exists of any amelioration in muscle activation in the elderly population.

*HIT* has gained popularity as a safe and efficient exercise method with the potential to influence several health-related parameters: in various populations, *HIT* has been shown not only to increase maximal oxygen consumption ($\dot{V}$O_2__max_) but also to decrease fat mass and increase insulin sensitivity (Gibala et al., 2006; Bruseghini et al., 2015). Moreover, it has been observed that *HIT* can lead to an increase of skeletal muscle oxidative and buffering capacity, muscle protein synthesis, and mitochondrial biogenesis (Scalzo et al., 2014). Despite the aforementioned data that suggest a potential positive impact of *HIT* on muscle remodeling and growth, there are limited data on the effect of *HIT* on muscle mass, strength, architecture, and quality in elderly (Astorino et al., 2011). *HIT* has been described to contrast the progressive loss of muscle mass (Harber et al., 2012) and 8 weeks of *HIT* were followed by the decrease of the percentage of adipose tissue and by the hypertrophic adaptation of the muscle involved in training (Bruseghini et al., 2015). If *HIT* induces also a substantial decrease of *IMAT*, this would result in a substantial improvement of muscle quality and function (Addison et al., 2014). In this regard, no clear data exist on the efficacy of *HIT* to reduce *IMAT* in elderly, active, healthy men, even though it has been shown that moderate physical activity (e.g., walking) was able to prevent the increase of *IMAT* in older subjects (Goodpaster et al., 2008).

On the basis of all these premises, we tested the hypothesis that muscular mass-volume, quality, morphology, and function can be increased/improved by both types of training intervention. Therefore, we evaluated the effects of 8 weeks of *HIT* and *IRT* on muscle quality, morphology, strength, and neuromuscular activation of healthy, elderly men. Also, we test the hypothesis that structural adaptations would differ between training modalities because of the differences in muscular and regional activation. The results would help to obtain a deeper insight into the specific effectiveness of these two types of training on muscular quality, morphology, and function in elderly subjects considering that these features have a substantial impact on the metabolic and locomotor roles of the skeletal muscle.

Therefore, we aimed to assess (i) whether *HIT* elicited any substantial increase of muscle strength and mass; (ii) whether muscle hypertrophy elicited by training was selectively more pronounced in specific areas of the quadriceps; (iii) if *HIT* and *IRT* were able to induce significant amelioration of the quality of the muscles and, in turn, modification of the strength-to-*ACSA* ratio; (iv) whether muscle morphology was substantially modified by the two training interventions; and (v) the relative contribution of muscle hypertrophy and muscular increased activation in eliciting the observed increments of strength.

## Materials and Methods

### Subjects

Twelve moderately active Caucasian men (*age*: 69.3 ± 4.2 years, range, 65–75; *body weight*: 77.8 ± 10.4 kg; *height*: 1.72 ± 0.05 m; *BMI*: 26.5 ± 2.8 kg m^–2^; *IPAQ score*: 4333 ± 1750 MET min week^–1^) were recruited through local advertisements in the Verona, Italy, metropolitan area and volunteered to participate in the study. Main physiological and health-related outcomes (e.g., $\dot{V}$O_2max_, body composition) have been previously published for this sample of participants (Bruseghini et al., 2015; Tam et al., 2018). All the subjects had filled in the IPAQ questionnaire and then they underwent a preliminary medical examination to evaluate exclusion criteria [abnormal *EKG* at rest and during exercise, uncontrolled hypertension, diagnosis of cardiovascular, respiratory and metabolic diseases, moderate-severe renal failure, neurological and orthopedic diseases limiting mobility and exercise, anti-coagulants and anti-aggregant therapy, alcohol and drug abuse and common contraindications to *MRI* (i.e., pacemakers, metallic clips)] and pathological responses to exercise. The study was conducted in accordance with ethical standards, the provisions of the Declaration of Helsinki, and national and international guidelines. The protocol and the methods of the study were approved by the Regional Review Board (approval on June 18, 2013), and written informed consent was obtained from each subject before entering the study.

### Experimental Design

A two-factor within-subject design was planned in which each subject received all the combinations of treatment that originated by crossing the two factors: one fixed factor was training modality (two levels, *HIT* and *IRT*); the second fixed factor was time (two levels, *Pre*- and *Post-*training) in which all subjects were exposed to all the two conditions (Keppel and Wickens, 2004). All the subjects were evaluated four times: before training for baseline values (*Pre*-*HIT*) and immediately after 8 weeks of *HIT* (*Post-HIT*), after 12 weeks of recovery before *IRT* (*Pre*-*IRT*), and, finally, after 8 weeks of resistance training (*Post-IRT*). Before the first data collection, a familiarization session was conducted, during which the experimental procedures were thoroughly explained and a simplified version of them was carried out.

During each experimental session, the tests were performed in the morning, at the same time of the day and in the environmentally controlled conditions, on three consecutive days: on the first day, the main anthropometric measurements were carried out and the $\dot{V}$O_2max_ of each subject was measured. The second day was devoted to functional strength test and ultrasound scan acquisition. The third day was dedicated to *MRI* scans. Twenty-four hours before the tests, participants abstained from strenuous physical activity and alcohol and caffeine consumption.

### Training Protocols

The subjects were asked to perform each supervised training session at the same time of the day on alternate days. Compliance to training was high with subjects in each training period completing all of the exercise sessions. No injuries or health disorders were reported during the exercise program, and no modification in the planned protocol had to be introduced. Between and during *HIT* and *IRT* sessions, the subjects were asked to maintain their habitual lifestyle: to evaluate physical activity, all subjects wore a portable monitor SenseWear Armband Mini (BodyMedia, Inc., Pittsburgh, United States) continuously for a 1-week period (Mackey et al., 2011). This has been done 1 month before each training period and during both training periods.

#### High-Intensity Interval Training

Volunteers trained three times a week for 8 weeks. Training consisted of seven 2-min bouts of cycling exercise (915 E, Monark, Varberg, Sweden) at 85–95% of individual $\dot{V}$O_2max_ interspersed by 2-min recovery intervals at about 40% of $\dot{V}$O_2max_ (Buchheit and Laursen, 2013a, b). Each series was preceded by 10 min of active warm-up. The entire supervised training session lasted from 45 to 50 min, including the post-training cooling-down phase. The mechanical workloads related to the percentage of $\dot{V}$O_2max_ were calculated using the individual oxygen consumption/load ratio of the warm-up before the incremental test and created using the oxygen consumption values measured in the last minute of each load. Heart rate/load (*HR/W*) ratio was also computed in order to control responses to exercise and to adjust workloads every 14 days, according to changes in the *HR/W* relationship assessed during three submaximal workloads at steady state.

#### Isoinertial Resistance Training

Resistance exercise was performed on a seated knee extension flywheel ergometer (Berg and Tesch, 1994) (YoYo Technology AB, Stockholm, Sweden) 3 times a week for 8 weeks. Each supervised session consisted of four sets of seven maximal, coupled concentric extensions and eccentric flexions of the knee from about 90° to 160°–170° knee joint angle. Subjects received verbal encouragement to push as harder as they could and direct feedback was provided during exercise by shoving force production. The increase in the maximum force applied during each training session has made it possible to enhance and adapt the workload constantly during the 8 weeks of training. The sets were interspersed by 3-min rest periods and initiated immediately after performing two submaximal actions. Each exercise session was preceded by 10 min of active warm-up, including three sets of seven submaximal actions with progressively increased effort. Training was performed using a polymer flywheel (4.2 kg). Each exercise session, including warm-up and rest periods, was completed in about 30 min.

### Anthropometry and Maximal Oxygen Uptake

Body weight and stature were measured and *BMI* was also calculated. $\dot{V}$O_2max_ was measured using a metabolic cart (Quark b^2^, Cosmed, Rome, Italy) at the end of incremental ramp tests to exhaustion on a cycle ergometer (Excalibur Sport, Lode, Groningen, Netherlands), as described in detail previously (Bruseghini et al., 2015).

### Muscle Volume, Cross-Sectional Area, and *IMAT*

To determine the volumes and *ACSA* of the total *quadriceps femoris (QF)*, *rectus femoris* (*RF*), *vastus lateralis* (*VL*), *vastus intermedius* (*VI*), and *vastus medialis* (*VM*), *MRI* scans in a 1.5-T GE scanner (General Electric, Milwaukee, WI) were obtained following the protocol described by Trappe et al. (2001). Briefly, a coronal scout scan [repetition time/echo time (TR/TE) 5 300/14 ms, field of view 48 cm, 256 × 160 matrix] of five slices 5 cm thick with 5-mm spacing was completed to establish orientation of the femur. Then, interleaved transaxial images of 1 cm thick (TR/TE 633/20 ms, field of view 274 × 480 mm, 256 × 256 matrix) were obtained along the entire length of the femur. The procedure has already been discussed previously (Bruseghini et al., 2015) and only the most salient details will be explained here. Analyses of the magnetic resonance images of dominant limb began with the first proximal slice not containing gluteal muscle and continued distally to the last slice containing *RF* (Castro et al., 1999). The average *ACSA* (cm^2^) was taken as the average of all the analyzed slices for an individual muscle and determined for the *RF*, *VL*, *VI*, and *VM* and summed for the total *QF*. *ACSA* was drawn manually in correspondence of slice obtained at 75, 50, and 25% of the length between the greater trochanter to the upper border of the patella (*LF*). The volume of muscle tissue per slice was calculated by multiplying the *ACSA* area by the inter-slice distance. The volumes of each of the *QF* components were calculated as the sum of all corresponding slice volumes. The volume of the quadriceps (*Vol*) was then computed as the sum of the single muscular volume.

*MRI* scans of dominant limb at 75, 50, and 25% femur length were examined to determine *IMAT* and subcutaneous adipose tissue using SliceOmatic image analysis software (version 4.2; TomoVision, Montreal, Quebec, Canada). *IMAT* was defined as adipose tissue area visible between quadriceps muscle groups. The gray-level intensity (threshold value) of the adipose tissue in the subcutaneous adipose tissue region was determined. This value was reduced by 20% to identify the quadriceps *IMAT* threshold (Rossi et al., 2010).

### Muscular Strength

Isometric and dynamic strength produced by the knee extensors of the dominant limb was evaluated with an isometric–isokinetic dynamometer (CMSi Cybex Humac Norm Dynamometer, Stoughton, MA, United States) at 90° of knee angle during maximal voluntary contraction in isometric condition (*T*_MVC_) and during concentric (*T*_C_) isokinetic contractions at an angular velocity of 120° s^–1^. Before the strength test, the subjects completed 10 min of warm-up exercise on a stationary bike, and they performed several practice trials while sitting on the reclining chair of dynamometer. The lower part of the leg was strapped to the end of the lever arm of the dynamometer and the center of rotation of the knee was aligned with the axis of the dynamometer. Three maximal trials (30 s of rest was provided between each trials) were performed for each condition with 3 min of recovery between each condition (Connelly and Vandervoort, 2000; Power et al., 2013). Visual feedback was provided to participants and verbal encouragement was standardized throughout both testing protocols (McNair et al., 1996). The highest torque values were recorded for further analysis.

### Muscle Architecture

Real-time B-mode ultrasonography (ACUSON P50 ultrasound system, 12L5 linear probe) was used to measure fascicle pennation angle (θ*_p_*) and fascicle length (*L*_f_) of the *VL*. Participants were sitting with the knee angle fixed at 90° (Raj et al., 2012). Images were obtained at mid-belly of the dominant *VL* muscle by using a linear-array probe. The probe was positioned perpendicular to the dermal surface of the *VL* muscle and oriented along the median longitudinal plane of the muscle. Mid-belly was defined as the point along the median longitudinal axis of the muscle at 50% of the distance between the proximal and distal apexes of the myotendinous junctions. The center of the probe was aligned to this position. The probe was coated with a water-soluble transmission gel to provide acoustic contact without depressing the dermal surface. Three images at rest were obtained within the same experimental session in each individual (Narici et al., 2003). Scans were analyzed with an open source software OsiriX (Pixmeo, Geneva, Switzerland). *VL* muscle thickness was defined as the distance between the superficial and deep aponeurosis. θ*_p_* was measured as the angle between the muscle fascicles and the deep aponeurosis. *L*_f_ was measured as the length of a fascicle between its insertions at the superficial and deep aponeurosis. Where the fascicles extended beyond the recorded image, *L*_f_ was estimated from muscle thickness (*T*_m_) and θ*_p_* using the following equation (Raj et al., 2012):

(1) L=fT×msinθp-1

Then, the *PCSA* of the quadriceps (*PCSA*) was calculated as follows (Gans, 1982):

(2) $$P\,C\,S\,A=V\,o\,l\times \theta_{p}\times L_{f}^{-1}$$

θ*_p_* and *L*_f_ measured on the *VL* were assumed to be representative of the mean θ*_p_* and *L*_f_ of the entire quadriceps (Erskine et al., 2009). Quadriceps specific tension (*T*_s_) was subsequently calculated using the following formula:

(3) $$T_{s}=T_{M}\,V\,C\,c\,o\,r\,r\times P\,C\,S\,A^{-1}$$

where *T*_MVCcorr_ is the tension in N calculated from *T*_MVC_ at 90° of knee flexion corrected by the patellar tendon moment arm length obtained from the literature (Smidt, 1973; Narici et al., 1992).

### Neuromuscular Activation

To determine the level of voluntary muscle activation and contractile proprieties of the dominant quadriceps muscle and biceps muscle of the arm (control condition), the interpolated twitch technique was used (Shield and Zhou, 2004). During experiments on lower limb, the participants sat in a standardized position with hip and knee at 90° of flexion, on a customized testing system’s chair and tightly secured to it with hip and torso straps. During experiments on upper limb, the elbow of the subjects was accommodated in a standardized position on a customized dynamometer placed on a table; the dominant forearm was then positioned vertically and connected to the load cell rigidly attached to the customized testing system. Electrical stimulation was administered via two 5 cm × 10 cm self-adhesive electrodes, placed distally (anode) and proximally (cathode) over the quadriceps (Kufel et al., 2002). The *quadriceps* and the *biceps brachii* were stimulated in a relaxed state with 50-mA pulses of 100 μs, which were increased in 30-mA increments (Digitimer High Voltage Stimulator model DS7A, Digitimer Ltd., Welwyn Garden City) until no further increase in twitch force was observed. This current was used 2 min later to elicit a single twitch during three maximal voluntary contractions (*MVC*) lasting 5 s each and a second twitch in the resting state 5 s after the *MVC*.

Force was measured by means of a calibrated load cell (DBBE, Applied Measurements Ltd., Aldermaston, Reading, United Kingdom) connected to a non-compliant strap that was placed around the subject’s dominant leg just superior to the ankle malleoli and around the forearm close to the wrist of the dominant arm. Torque signals and electrical stimuli collected with the help of PowerLab data acquisition (PowerLab 16/35 AD Instruments Ltd., Australia) at a sampling frequency of 1 kHz and analyzed by Labchart 6.0 software (AD Instruments Ltd., Australia).

Voluntary activation of the stimulated muscles (*%Act*) was quantified applying the following equation:

(4) %Act=[1-(superimposedtwitch/controltwitch)]×100

where the superimposed twitch is the force increment noted during a maximal voluntary contraction at the time of stimulation and the control twitch is that evoked in the relaxed muscle (Shield and Zhou, 2004).

### Statistical Analysis

All values in the text are presented as mean ± standard deviation. Sample size was determined using G^∗^Power software (ver 3.1.9.2) (Faul et al., 2007) to ensure there was sufficient power (1–β = 0.80) to detect significant differences within factors. Normality of data distribution was evaluated by means of the Shapiro–Wilk test (StatPlus: mac Version v6, AnalystSoft, CA, United States). When criteria for normality were not met, inferential analysis was always performed on the log-transformed data. Overall analysis of the data was carried out according to Keppel and Wickens (2004) for two-fixed factors within-subjects design with Training type (*HIT* and *IRT*) and Time (*Pre* and *Post*) as fixed. In particular, (i) *F* values were calculated taking into account the possible violation of sphericity by using the correction of the degree of freedom, as suggested by Geisser and Greenhouse; (ii) effect size in terms of population variability was evaluated by computing ω*^2^*, which expresses the variability of the effect over the sum of that variability and the error variability and total variability that affects it; (iii) single planned contrasts time (*Pre* vs. *Post*) and Training modalities, *HIT* vs. *IRT* were evaluated; (iv) effect size (*d*) of the differences between the contrasted values was also calculated. Calculations were carried out using an Excel spreadsheet (MO 2010, Microsoft Corp. Seattle, WA, United States) programed for this purpose.

The differences between the percent increase of the cross-sectional areas of the quadriceps between *Pre*- and *Post*-interventions were evaluated by using a 2-ANOVA analysis for repeated measurements; *post*-*hoc* analysis was carried out between families of pairwise comparisons by using the Šidák–Bonferroni procedure to correct for the family-wise Type I error. Multiple linear regressions between a dependent variable and two independent explanatory variables were calculated by using least squares approach (Motulsky and Christopoulos, 2004).

Correlation analyses were conducted on *ACSA*, *IMAT*, %*Act*, with Pearson’s product–moment correlation, and correlation coefficients (*r*) were classified as small (0.1 < *r* ≤ 0.3), moderate (0.3 < *r* ≤ 0.5), high (0.5 < *r* ≤ 0.7), very high (0.7 < *r* ≤ 0.9), and almost perfect (*r* > 0.9) (Hopkins et al., 2009).

## Results

The results have been reported synthetically (mean ± SD) in Supplementary Table S1 for greater clarity and commented in the following sessions.

### Cross-Sectional Areas

**FIGURE 1:**
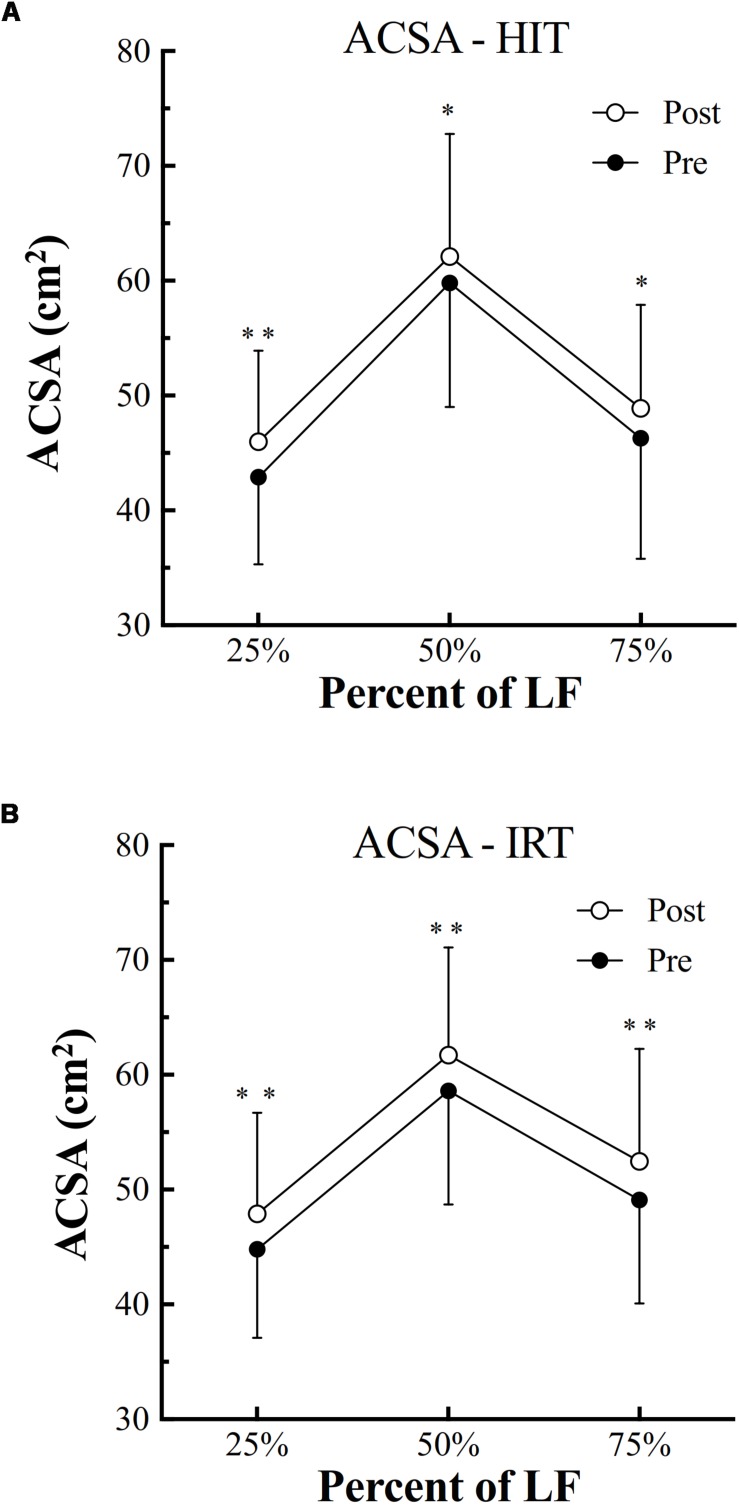
Anatomical cross-sectional area (*ACSA*, cm^2^) of the quadriceps assessed before (filled symbols) and after (empty symbols) high-intensity interval training (*HIT*, **A**) and isoinertial resistance training (*IRT*, **B**) at 75, 50, and 25% of the length between the greater trochanter to the upper border of the patella (*LF*) (^∗^*P* < 0.05; ^∗∗^*P* < 0.01).

*HIT* was followed by a significant increase of *ACSA* at all the three femur lengths [at 25%: plus 3.09 cm^2^ ± 1.38; (*P* = 0.001; 95% CI of diff: 2.21–4.0; *d*: 2.24); at 50%: plus 2.27 cm^2^ ± 2.52 (*P* = 0.010; 95% CI of diff: 0.67–3.87; *d*: 0.90); at 75%: plus 2.65 cm^2^ ± 3.04 (*P* = 0.011; 95% CI of diff: 0.72–4.58; *d*: 0.87)] (Figure 1).

Also, *IRT* was followed by a significant increase of *ACSA* at all the three evaluated % of femur length [at 25%: plus 3.19 cm^2^ ± 1.24; (*P* = 0.001; 95% CI of diff: 2.40–3.99; *d*: 2.57); at 50%: plus 3.03 cm^2^ ± 3.04 (*P* = 0.005; 95% CI of diff: 1.10–4.96; *d*: 1.00); at 75%: plus 3.40 cm^2^ ± 3.21 (*P* = 0.004; 95% CI of diff: 1.36–5.44; *d*: 1.06)] (Figure 1).

ANOVA analysis revealed a significant effect of factor *Time* on the increase of *ACSA* at 25% of femur length (*F* = 0.001; ω*^2^* = 0.59), 50% of *LF* (*F* = 0.001; ω*^2^* = 0.64), and 75% (*F* = 0.002; ω*^2^* = 0.25). At 25% and at 75% of femur length, also an effect of factor *Training* was present (*F* = 0.028; ω*^2^* = 0.10): at 25%, *ACSA* at *Post*-*IRT* was larger than at *Post*-*HIT* (*P* = 0.024; *d* = 0.753); at 75%, *ACSA* at *Post*-*IRT* was larger at *Post*-*HIT* (*P* = 0.008; *d* = 0.929) than at *Pre*-*HIT* (*P* = 0.011; *d* = 1.291). In none of the cases, however, significant interactions between factors were present.

The highest quadriceps *ACSA* was found at 50% of femur length. However, for both trainings, the percent increases of quadriceps *ACSA* at the 3% of femur length were rather homogeneous and they were not significantly different. The percent increases of quadriceps *ACSA* at the 3% of femur length were not different between *HIT* and *IRT*.

Finally, the percent increase of quadriceps *ACSA* observed after trainings at the three levels of femur length was negatively and moderately related with the absolute area at *Pre*-*HIT* and *Pre*-*IRT*: *r* = -0.37; *P* = 0.001; *n* = 72.

### Volume of the Quadriceps

**FIGURE 2:**
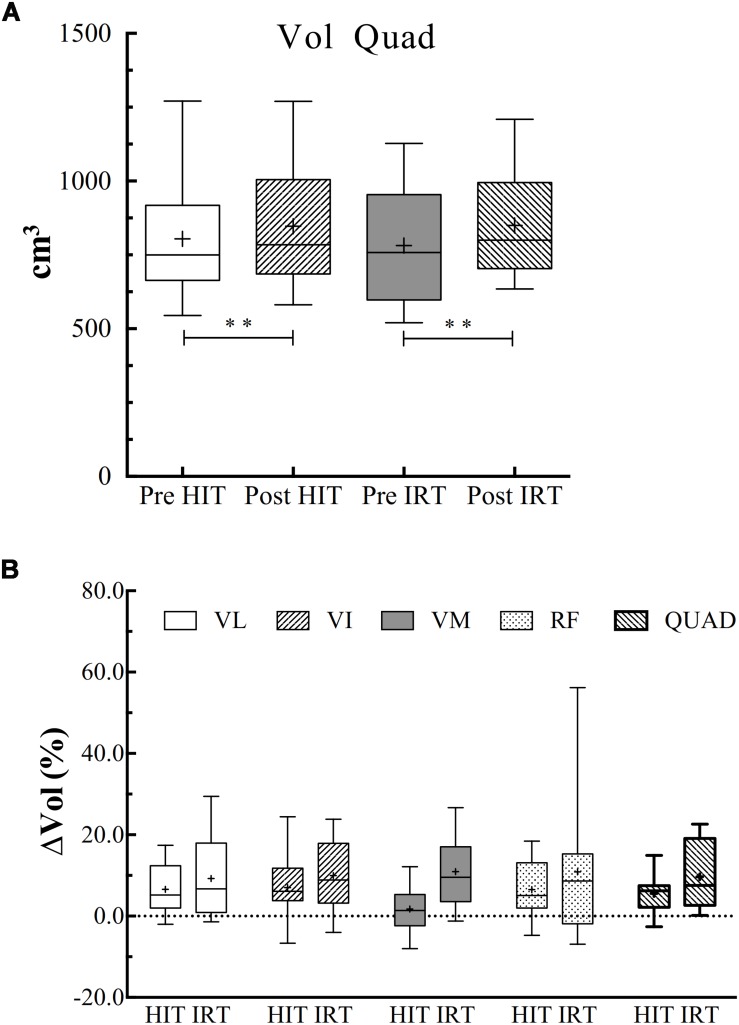
**(A)** Box and whiskers plot of the volumes of the quadriceps (*Vol*, cm^3^) before and after *HIT* and *IRT*. Asterisks indicate the significant difference between *Pre*- and *Post*-conditions (^∗∗^*P* < 0.01). **(B)** Box and whiskers plots of the percent increases (*Vol*, %) of the entire quadriceps and of each of its belly observed during *HIT* and *IRT*. The box extends from the 25th to 75th percentiles. The line in the middle of the box is plotted at the median and “+” at the mean. Whiskers range from a Min to Max value.

ANOVA analysis revealed a significant effect of time on the increase of *Vol* (*F* = 0.001; ω*^2^* = 0.848). *Vol* was significantly larger at *Post*-*HIT* than at *Pre*-*HIT*: plus 42.2 cm^3^ ± 38.3 (*P* = 0.003; *d* = 1.11), and at *Post*-*IRT* than at *Pre*-*IRT*: plus 68.2 cm^3^ ± 38.3 (*P* = 0.001; *d* = 1.40) (Figure 2).

The percent increase of the total *Vol* of the quadriceps was not significantly different between *HIT* and *IRT*.

### Intermuscular and Subcutaneous Adipose Tissues

ANOVA analysis on the data of *IMAT* at 50% of femur length was performed after log transformation of the data as they failed the test for assessing normal distribution. The analysis revealed a significant effect of time (*F* = 0.001; ω*^2^* = 0.78) and of training (*F* = 0.001; ω*^2^* = 0.66).

**FIGURE 3:**
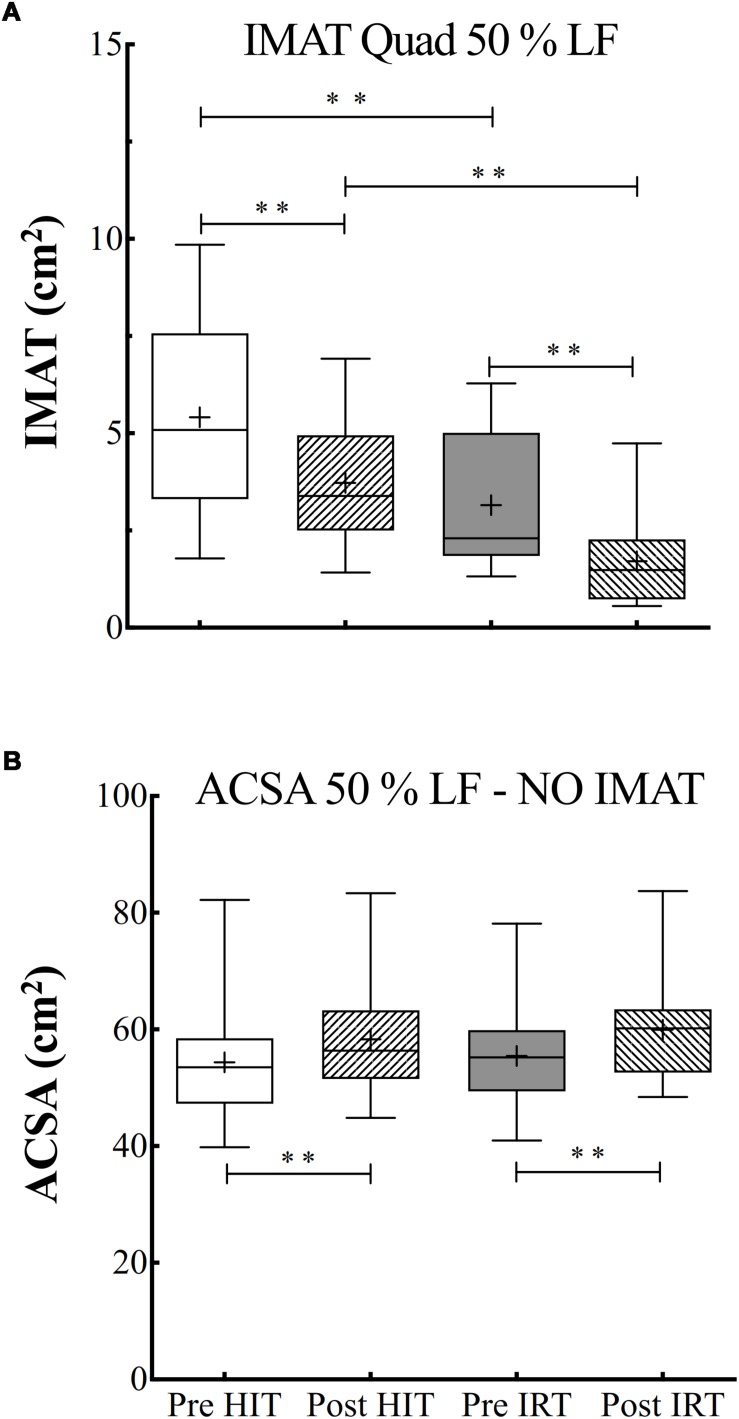
**(A)** Box and whiskers plots of intermuscular adipose tissue (*IMAT*, cm^2^). **(B)** Box and whiskers plots of *ACSA* (cm^2^) measured at 50% of the length between the greater trochanter to the upper border of the patella (% *LF*) excluding *IMAT*. Lines and asterisks indicate the significant differences between the mean values (^∗∗^*P* < 0.01). The box extends from the 25th to 75th percentiles. The line in the middle of the box is plotted at the median and “+” at the mean. Whiskers range from a Min to Max value.

There was a progressive decrease of the *IMAT* area during the study. In particular, *IMAT* was significantly lower at *Post*-*HIT* than at *Pre*-*HIT* (*P* = 0.001; *d* = 1.57). As the analysis was performed on log-transformed data, the 95% CI of the ratio between the *Pre*-*HIT* and *Post*-*HIT* were calculated: 0.70–1.15. *IMAT* significantly decreased also after *IRT* in respect to *Pre*-*IRT* (*P* = 0.003; *d* = 1.08), the 95% CI of the ratio being equal to 0.51–1.49. *IMAT* after the strength training was also significantly smaller than at *Post*-*HIT* (*P* = 0.001; *d* = 1.64) with 95% CI of the ratio equal to 0.42–1.40) (Figure 3).

Also, for subcutaneous adipose tissue at 50% of femur length, it was possible to show an effect of time (*F* = 0.001; ω*^2^* = 0.63): subcutaneous adipose area significantly decreased after *HIT* in respect to the initial control condition (*P* = 0.001; *d* = 1.31, 95% CI of diff: –5.97 to –7.73). An effect of the type of training was also evident (*F* = 0.001; ω*^2^* = 0.66). In addition, significant interactions between time and training effects were also demonstrated (*F* = 0.003; ω*^2^* = 0.56), as the differences between *Pre*-*HIT* and *Post*-*HIT* values were significantly different from those observed before and after *IRT* (*P* = 0.010; *d* = 0.99).

The absolute and percent changes of *ACSA* at 50% of femur length were also calculated net of *IMAT* contribution. The subtraction of *IMAT*, however, did not alter the pattern of the changes observed after the two training interventions: *ACSA* at *Post*-*HIT* was significantly larger than at *Pre*-*HIT*: plus 3.95 cm^2^ ± 3.17 (*P* = 0.001; *d*: 1.25; 95% CI of diff: 1.93–5.96) and at *Post*-*IRT* than at *Post*-*IRT*: plus 4.47 cm^2^ ± 2.63 (*P* = 0.001; *d*: 1.697; 95% CI of diff: 2.79–6.14). Interestingly, the percent increases of total *ACSA* neglecting *IMAT* were significantly larger than the ones measured including *IMAT* both for *HIT* (7.71% ± 6.21 vs. 4.00% ± 4.29; *P* = 0.001, 95% CI of discrepancy from 0: 2.1–5.3) and for *IRT* (8.38% ± 5.47 vs. 5.65% ± 6.14; *P* = 0.006, 95% CI of discrepancy from 0: 0.97–4.48) (Figure 3).

For the sake of completeness, also the analysis of *IMAT* at 25% and at 75% of femur length was carried out after log transformation of the data. The data generally confirmed the ones obtained at 50%: the analysis revealed a significant effect of time (*F* = 0.001; ω*^2^* = 0.70) at 25% of femur length. In details, *IMAT* was significantly lower at *Post*-*HIT* than at *Pre*-*HIT* (*P* = 0.003; *d* = 1.12) and at *Post*-*IRT* than at *Pre*-*IRT* (*P* = 0.008; *d* = 0.92). At 75% of femur length, however, *IMAT* turned out to be significantly lower only after *IRT* than at *Pre*-*IRT* (*P* = 0.001; *d* = 1.244).

Finally, the net decrement of *IMAT* observed in the two training interventions was highly correlated (*r* = –0.71) with the initial absolute value of *IMAT*.

### Muscle Torque

**FIGURE 4:**
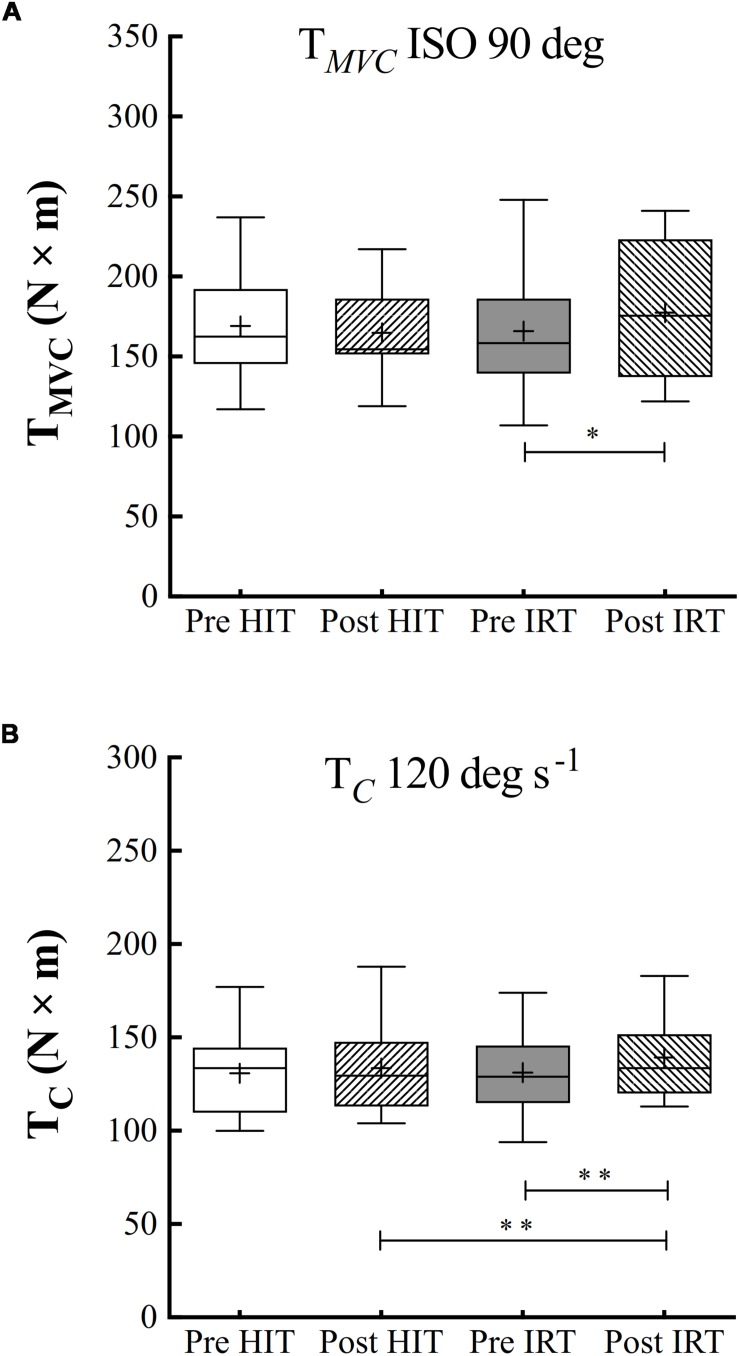
Box and whiskers plots of isometric torque at 90° of knee flection **(A)** and isokinetic concentric torque (T*_C_*, N × m) at 120° s^–1^ of angular velocity **(B)**. Lines and asterisks indicate the significant differences between the mean values (^∗^*P* < 0.05; ^∗∗^*P* < 0.01). The box extends from the 25th to 75th percentiles. The line in the middle of the box is plotted at the median and “+” at the mean. Whiskers range from a Min to Max value.

Only *IRT* training was followed by a significant increase of *T*_MVC_ measured at 90° of knee joint flexion: plus 11.5 N m ± 17.1 (*P* = 0.040; *d* = 0.67): the percent of increase amounted to 7.0% ± 9.8 (Figure 4).

Training significantly affected *T*_C_ at 120° s^–1^ (*F* = 0.014; ω*^2^* = 0.137), but isokinetic strength was only significantly larger after *IRT* than before *IRT*: plus 8.8 N m ± 13.0 (*P* = 0.008; *d* = 0.93). For strength values, the baseline conditions *Pre*-*HIT* are comparable to baseline conditions *Pre*-*IRT* (Figure 4).

Percent increases of *T*_C_ were not linearly related with the corresponding initial torque values (*P* between 0.050 and 0.705). They ranged, on average, from 2.44 to 10.4% (grand mean at 120° s^–1^: 6.6% ± 11.3).

### Pennation Angle, PCSA, and Specific Torque

**FIGURE 5:**
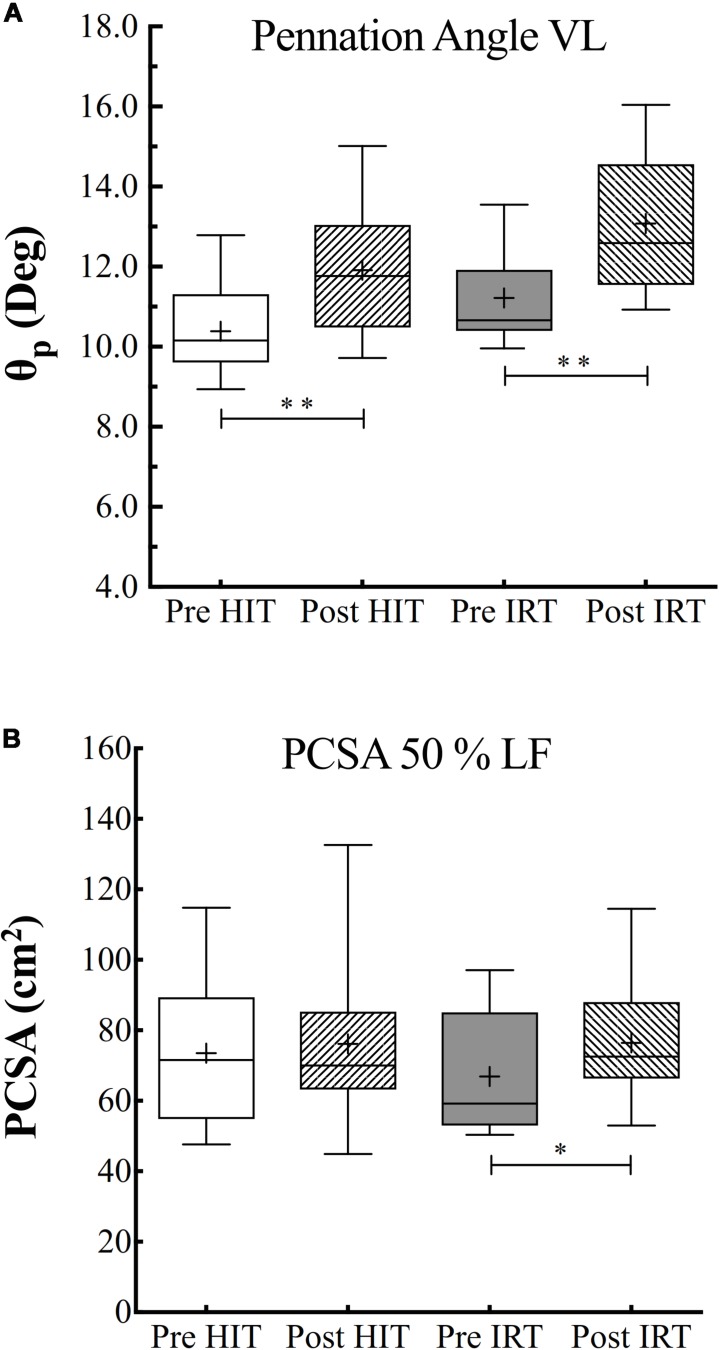
Pennation angle (θ*_p_*, °) of the fibers of *vastus lateralis* **(A)** and physiological cross-sectional area (*PCSA*) of quadriceps in cm^2^ **(B)** assessed before (*Pre*) and after (*Post*) *HIT* and *IRT*. Lines and asterisks indicate the significant differences between the mean values (^∗^*P* < 0.05; ^∗∗^*P* < 0.01). The box extends from the 25th to 75th percentiles. The line in the middle of the box is plotted at the median and “+” at the mean. Whiskers range from a Min to Max value.

ANOVA analysis revealed a significant effect of time (*F* = 0.019; ω*^2^* = 0.410) and of training (*F* = 0.001; ω*^2^* = 0.119) on θ*_p_*, which turned out to be significantly greater after *HIT* than before (*P* = 0.001; *d* = 1.93) and after *IRT* than before strength training (*P* = 0.004; *d* = 1.03) (Figure 5).

*PCSA* at 50% of femur length was significantly affected by training (*F* = 0.041; ω*^2^* = 0.083), but was only larger at *Post*-*IRT* than at *Pre*-*IRT* (*P* = 0.025; *d* = 0.741) (Figure 5).

**FIGURE 6:**
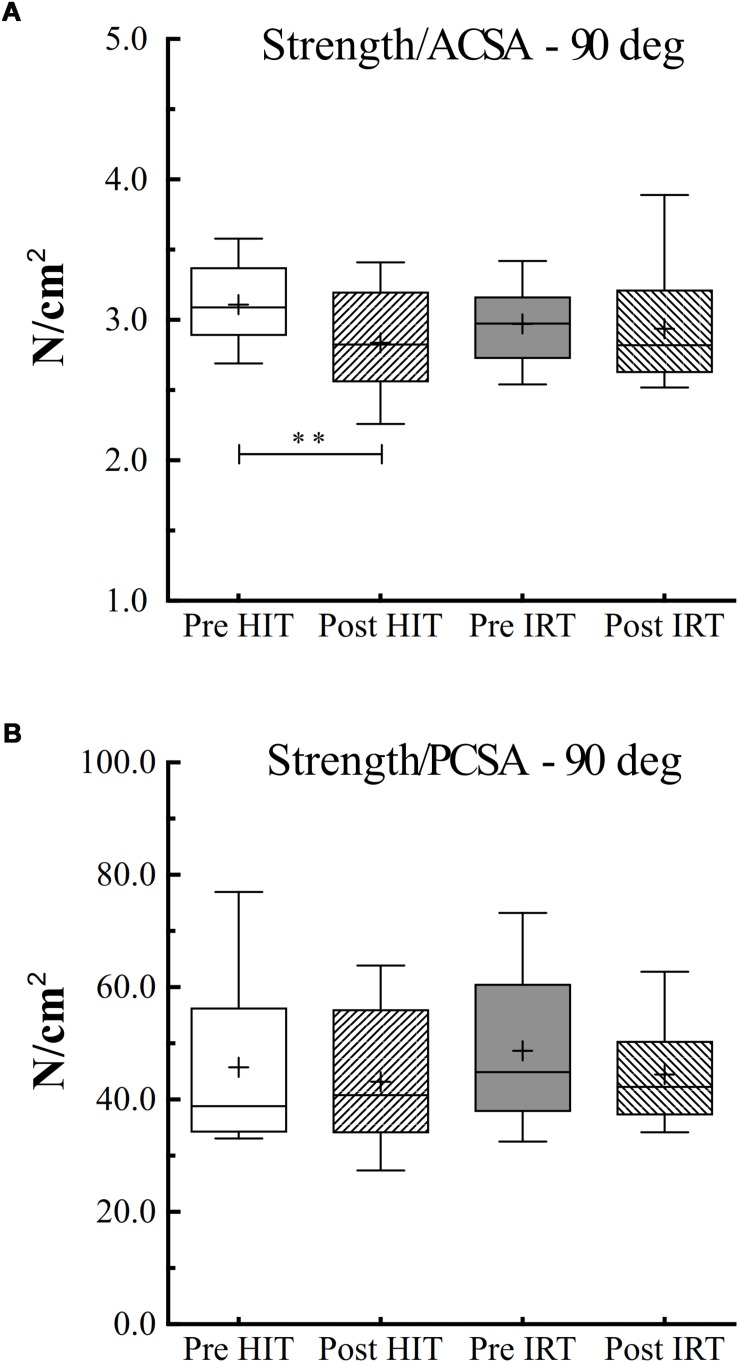
Box and whiskers plots of specific isometric strength (90° of knee flection) per unit of area of *ACSA* **(A)** and *PCSA* **(B)** in N cm^–2^. Lines and asterisks indicate the significant differences between the mean values (^∗∗^*P* < 0.01). The box extends from the 25th to 75th percentiles. The line in the middle of the box is plotted at the median and “+” at the mean. Whiskers range from a Min to Max value.

However, when specific isometric strength was calculated as the ratio between strength and *ACSA*, we obtained a different pattern if we corrected or did not correct *ACSA* for *IMAT* (Figure 6). When *IMAT* is included, torque per square centimeter of *ACSA* did not change during the study, and its grand average amounted to 59.8 N cm^–2^ ± 7.1, 95% CI 61.8–57.7. When *ACSA* was corrected for the contribution of *IMAT*, torque per squared cm of *ACSA* at *Post*-*HIT* (60.8 N cm^–2^ ± 7.5) was smaller than at *Pre*-*HIT* (66.4 N cm^–2^ ± 6.1), *P* = 0.007, 95% CI of the difference from 0: 1.9–9.3 N cm^–2^. Conversely, it did not change from *Pre*-*IRT* (63.8 N cm^–2^ ± 5.6) to *Post*-*IRT* (63.0 N cm^–2^ ± 9.1). Finally, specific strength measured as the ratio between *T*_MVC_ and *PCSA* remained constant during the study, and its grand average amounted to 45.5 N cm^–2^ ± 12.0, 95% CI 49.0–42.0.

### Muscular Activation

**FIGURE 7:**
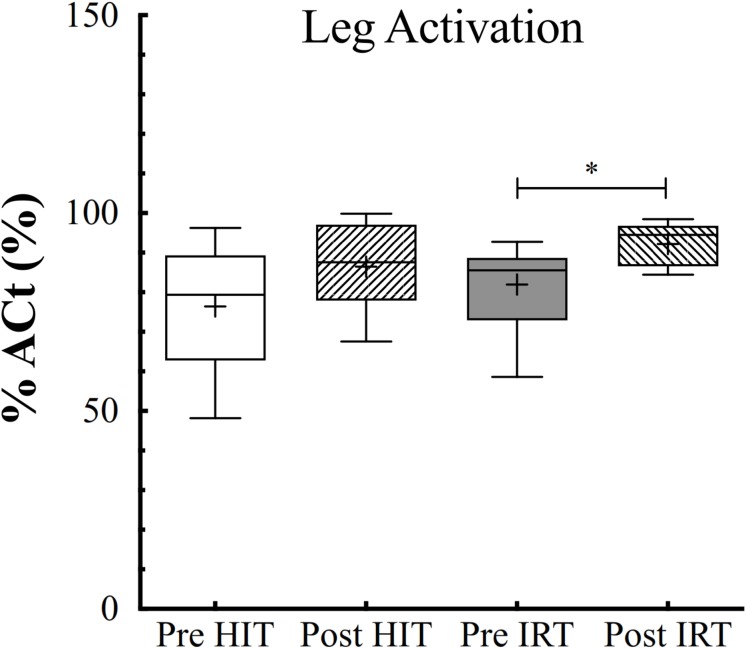
Box and whiskers plots of percent of voluntary activation (*%Act*, %) of the lower limb extensors assessed before (*Pre*) and after (*Post*) *HIT* and *IRT*. Lines and asterisks indicate the significant differences between the mean values (^∗^*P* < 0.05). The box extends from the 25th to 75th percentiles. The line in the middle of the box is plotted at the median and “+” at the mean. Whiskers range from a Min to Max value.

ANOVA analysis for the two fixed effects demonstrated that %*Act* of the quadriceps was affected by training (*F* = 0.019; ω*^2^* = 0.119), being larger at *Post*-*IRT* than at *Pre*-*IRT* (*P* = 0.011; *d* = 0.897) (Figure 7). Arm activation remained identical throughout the study.

Moreover, %*Act* of the quadriceps was negatively correlated with the absolute levels of *IMAT* both before (*r* = 0.21, small correlation) and after (training (*r* = 0.53, high correlation).

The percent increases of isokinetic and isometric torque selected as explanatory variables (Y) were fitted to a multiple linear regression with the percent increases of *ACSA* at 50% of femur length (X_1_) and of %*Act* (X_2_) as predictive variables for both *HIT* and *IRT* conditions, forcing the functions to pass through the origin of the axis:

(5a) $$\begin{array}{c} H\,I\,T:Y=X_{1}\times 0.568\,\left(0.252\right)+X_{2}\times 0.041\,\left(0.037\right);\\ (F=3.78;P=0.03 \end{array}$$

(5b) $$\begin{array}{c} I\,R\,T:Y=X_{1}\times 0.634\,\left(0.209\right)+X_{2}\times 0.152\,\left(0.075\right);\\ \left(F=9.45;P=0.0001\right); \end{array}$$

where Y, X_1_, and X_2_ represent the percent increases of torque, *ACSA*, and %*Act*, respectively.

However, in *HIT*, only the percent of increase of *ACSA* significantly predicted the increase of strength (*t* = 2.25; *P* = 0.03); in *IRT*, both percent of increases of *ACSA* (*t* = 3.03; *P* = 0.004) and %*Act* (*t* = 2.031; *P* = 0.048) significantly predicted the percent increase of Torque.

## Discussion

The purpose of this investigation was to compare the effects of *HIT* and *IRT* on muscular strength, mass, morphology, muscle quality, i.e., *IMAT*, and neuromuscular activation in a group of active, healthy elderly subjects.

The main findings showed that:

i) *T*_c_ and *T*_MVC_ turned out to be significantly larger only after *IRT*; *ACSA* of quadriceps significantly increased both after *HIT* and after *IRT* at three evaluated femur lengths; the efficacy of training was also confirmed by the analysis of the changes of *Vol*;
ii) *IMAT* at 50% of femur length decreased after both trainings, in particular after *IRT*, and moreover, although the removal of *IMAT* did not change the pattern of the percent change of *ACSA* after trainings, it turned out to be larger than the ones calculated including *IMAT* area;
iii) θ*p* increased both after *HIT* and *IRT*; however, *PCSA* turned out to be larger only after *IRT*; the changes in *PCSA* and in torque resulted in constant value of specific strength per unit of *PCSA*;
iv) *%Act* of the quadriceps was only greater after the *IRT* and the increments of strength observed after *IRT* seem to be predicted by the consensual increases of *ACSA* and %*Act*.

Therefore, both *HIT* and *IRT* seemed to be able to induce significant and remarkable changes in muscle mass, morphology, and quality, but strength turned out to be significantly and positively affected only by *IRT*. The overall analysis of physical activity during the intervention period shows that subjects were able to maintain the same lifestyle, and they have not changed their *total daily energy expenditure* during the two training programs (before *HIT*: 2318 ± 315 kcal/day; during *HIT*: 2255 ± 314 kcal/day; before *IRT:* 2253 ± 243 kcal/day; during *IRT*: 2344 ± 220 kcal/day).

### Muscle *ACSA* and Volume

Both *HIT* and *IRT* were able to induce muscle hypertrophy, since *ACSA* increased at all the three femur lengths, as shown in Figure 1, and *Vol* was augmented after the two training interventions (Figure 2). The findings after *HIT* are in agreement with the ones reported by other investigators (Harber et al., 2012; Estes et al., 2017) who showed a significant increase of *Vol* in older and young men after moderate-to-vigorous aerobic training. The percent increase reported in those studies is of the same order of magnitude as the one found in the present investigation, i.e., 5.5% ± 4.6. Harber et al. (2012) attributed the observed hypertrophy to the specific increase of *ACSA* of *MHC Type 1* fibers.

Strength training has been shown to induce remarkable increases of muscle mass in elderly subjects (Cadore et al., 2014) and 10 to 12 weeks of heavy resistance training resulted in increase of *ACSA* of the quadriceps ranging from 6% (Kraemer et al., 1999) to 11–14% (Häkkinen et al., 2000). In the present study, the average percent increase of *ACSA* at 50% of *LF* was 5.7% ± 6.4, a value that might be explained by the shorter duration of the intervention and by the different training protocols applied in the studies. In this respect, *IRT* with eccentric overload has been claimed to be more effective and capable to induce larger gains in muscle mass and strength (Maroto-Izquierdo et al., 2017) than traditional weight lifting. However, a recent review (Vincens-Bordas et al., 2018) has refuted this conclusion, highlighting that the available data do not allow us to conclude that *IRT* is superior to traditional weight lifting and gravity-dependent strength training in increasing muscle mass and strength. Yet, it is worth noting that the majority of the available data were collected on young or adult men and women and conclusive data concerning elderly individuals on the comparison of the two training modalities are scarce.

The largest *ACSA* was found at 50% of femur length in agreement with the findings of Narici et al. (1996). However, in contrast with their findings, and with the ones of Mangine et al. (2018), the increase of *ACSA* found after the two trainings was uniform at the three levels of femur length. The discrepancy between the data obtained in this investigation and the ones reported in the quoted paper deserves, of course, a comment. First, our study concerned elderly subjects, whereas others investigated adult young volunteers. We know that different increases of *ACSA* have been found between young and elderly subjects (Kraemer et al., 1999), although this has not been consistently confirmed (Cadore et al., 2014). Therefore, we may speculate that the less pronounced response of the elderly subjects to the training stimulus may have somehow blunted and made less evident the selective growth of the four bellies of the quadriceps. Secondly, *IRT*, with its eccentric overload, may lead to a massive activation of all the bellies of the quadriceps, thus overriding the limitation of the gravity-dependent training that may induce activations of different magnitude as a consequence of the different loads imposed to each singular muscle by their mechanical gains (Folland and Williams, 2007).

Muscular morphological adaptations are also paralleled by substantial changes in muscle architecture. There is a general agreement that the pennation angle (θ*_p_*) of the muscle fibers increases with hypertrophy (Kawakami et al., 1993; Folland and Williams, 2007). The increase of θ*_p_* would allow a larger packing of fibers for the same *ACSA* and lead to the increase of the *PCSA*, i.e., the area perpendicular to the line of application of the force produced by the fibers. However, an increase of θ*_p_* would bring about a decrease of the force applied to the tendon because the angle between the fibers and the line along which the force is projected decreases. Yet, it can be geometrically demonstrated that if θ*_p_* stays below 45°, its increase is compensated by the increase of *PCSA* so that an augmentation of force results after training (Alexander and Vernon, 1975). Indeed, it has been shown that an increase of θ*_p_* from 8.0° to 10.7° (+36%) increased *PCSA* and force (+16%) more than *ACSA* (+10%) (Aagard et al., 2001).

### Intermuscular Adipose Tissue

Many physiological/pathological conditions (e.g., aging, sedentary lifestyle, but also the augmentation of inflammatory cytokines, reduced anabolic hormonal response, and general metabolic disorders) lead to muscle deconditioning, a phenomenon characterized by a loss of muscle strength and power and an increase of fatty infiltration. Data suggest that *IMAT* may be a prominent indicator to track metabolic-dependent activity and skeletal muscle quality. The *IMAT* increase and accumulation are linked to muscle dysfunction and is largely attributable to inactivity, but it is also associated with increasing age (Marcus et al., 2010). *IMAT* negatively affects muscle quality and muscle function by decreasing absolute and specific strength levels leading to muscle weakness. The latter have been associated with high levels of *IMAT* in adults with other comorbidities (Marcus et al., 2010) and/or characterized by low levels of physical activity (Addison et al., 2014).

The efficacy of resistance training to decrease *IMAT* in adult and elderly subjects has already been documented (Nicklas et al., 2015), although the intensity of the training remains crucial: no decrease in thigh *IMAT* occurs if the eccentric intervention is carried out at submaximal intensity (Jacobs et al., 2014). Less clear and definitive data exist on the efficacy of endurance training, let alone *HIT*, to abate *IMAT* infiltration in healthy elderly subjects. The benefits of low-intensity endurance training have been evaluated (Ikenaga et al., 2017); yet, the effectiveness of *HIT* is still unknown. Notwithstanding that the present study has been conducted on a small number of subjects, the data suggest that even short periods of high-intensity aerobic training may be effective to reduce *IMAT* in elderly healthy subjects.

The improvement in the muscle quality was also highlighted by finding that the percent increases of *ACSA* obtained when *IMAT* was neglected were significantly larger than the ones obtained when *IMAT* was included in the planimetric calculation of *ACSA*.

It has been suggested that only individuals with a low infiltration of *IMAT* are able to significantly ameliorate their muscle quality (Marcus et al., 2010). In the present study, however, we observed a negative and very high correlation (*r* = 0.71) between the *IMAT* content before the training and the net decrease of *IMAT* observed after *HIT* and *IRT*. The regression analysis also implied that the two training modalities were able to induce an average percent decay of *IMAT* of about 25% (95% CI 51.2-1.3). These results highlight the efficacy of *HIT* and *IRT* training programs in reducing the contribution of non-functional *IMAT.*

### Muscular Strength, Architecture, and Activation

Muscle strength significantly increased only after *IRT*. This type of training has been found to be effective for increasing muscular performances of the limb extensors in elderly people (Onambélé et al., 2008), and the average percent increase of *T*_MVC_ found in the present investigation (7.0% ± 9.8) is close to the one reported in the quoted paper. One unexpected finding consisted in the absence of any substantial and significant increase of strength after *HIT*, in spite of documented increases of *ACSA* and *Vol*. The dissociation between functional and morphological adaptations requires some extended comment.

As we know, the increase of muscular strength is a functional adaptation that results from several morphological and neural mechanisms that intervene during training (Folland and Williams, 2007). In this regard, the changes of muscular architecture are of paramount importance in order to understand how they may affect the improvement of strength consequent to the increase of muscle mass. As we have already outlined, there is a large bulk of evidence that shows that the θ*_p_* of muscle fibers is increased in strength-trained muscles (Kawakami et al., 1993) and it augments with muscle hypertrophy (Aagard et al., 2001). This morphological change is beneficial because it will allow a greater packing of fibers for the same *ACSA* increasing *PCSA*, i.e., the area normal to the line of application of the force produced by the fibers. Of course, an increased θ*_p_* will also decrease the force applied by the muscle along the tendon. Therefore, there is a sort of trade-off between the increase of muscle mass and the widening of θ*_p_* on the strength measured at the ending of the tendon. Even though it has been geometrically demonstrated that any increase of θ*_p_* that stays below 45° is compensated by the increase of *PCSA*, wherefrom an increase of strength, however, occurs, in the present case, the increase of θ*_p_* after *HIT* was not probably sufficient to induce a substantial augmentation of *PCSA* (Figure 5). We must also consider that *PCSA* of the quadriceps was calculated by assuming an identical angle of θ*_p_* measured at the level of the *VL*, which, in turn, was considered representative of the mean θ*_p_* of the entire quadriceps. Of course, this assumption may have introduced an unpredictable error in the calculated *PCSA* of the quadriceps.

Secondly, the level of neuromuscular activation increased only after *IRT* in respect to the control condition before strength training. Even admitting that the interpolated twitch technique is not freed of several technical and methodological issues (Folland and Williams, 2007) and that activation is muscle specific (Belanger and McComas, 1981) and angle specific (Becker and Awiszus, 2001), one must acknowledge that recent studies confirm that strength training is followed by the increase of muscular activation in elderly humans (Reeves et al., 2004). Flywheel resistance training elicits a greater muscle activation of the involved muscles during isometric maximal voluntary contraction; in addition, muscular activation during both eccentric and concentric contraction seems to be maximal even in the early phases of training over the entire range of movement (Norrbrand et al., 2010). The capability of flywheel resistance training of inducing a greater neuromuscular activation may well explain the findings, after *IRT*, of a higher level of activation of the trained quadriceps. By incidence, the empirical model proposed by calculating the multiple linear regression between the percent increases of isokinetic torque and the percent increases of *ACSA* at 50% of femur length (X_1_) and of %*Act* (X_2_) seems to suggest that the gain in strength achieved with *IRT* was more ascribed to the increase of muscle mass than to the amelioration of muscle activation.

*HIT* was not followed, though, by any substantial increase of neuromuscular activation. It has been shown that, during cycling, the four bellies of the quadriceps are activated with different timing: *VL* and *VM* show the highest activation in the pushing phase from the top dead center to about 90°, or the first quadrant of the cycle; *RF*, a bi-articular muscle, shows bursts of biphasic activation in the first (0°–90°) and fourth (270°–360°) quadrants (Lima da Silva et al., 2016). We can therefore suggest that flywheel training seems to be able to induce greater neuromuscular activation of the involved muscles along the entire range of motion in respect of cycling and, hence, contribute substantially to the increase of strength.

## Limitations and Strengths

The investigation is not freed from methodological weaknesses. The primary limitation to the generalization of our results is the absence of a control group: considering the experimental design, the study was not counterbalanced. As such, it suffered from intrinsic limitations, since the results were not freed from incidental effects other than the one directly induced by the interventions, and a longer washout period between training sessions could be considered in future studies. Furthermore, the effects of training on the antagonist muscles or on all the thigh muscles could be evaluated.

In this investigation, we evaluated for the first time the effect of *HIT* and of *IRT* on the *IMAT* and muscle quality of the quadriceps in elderly subjects. In order to unveil which changes occurred in muscle tissue due to these types of trainings, a group of elderly, although healthy, untrained volunteers were investigated, since, in this category of subjects, an age-related impaired muscular response has been frequently described. Despite initial doubts about the feasibility of carrying out high-intensity workouts with elderly subjects, we found that *HIT* and *IRT*, when performed with care and customizations, are absolutely safe and well tolerated by the subjects. Therefore, the meaning and the applicability of the results obtained in this study may be relevant to address training interventions in elderly subjects: our study protocol has been applied on healthy subjects; however, the effects of *HIT* and *IRT* on *IMAT* must also be evaluated in sarcopenic subjects.

## Conclusion

Although these results must be interpreted with caution and the limitations of the study should be borne in mind, our results indicate that *HIT* and *IRT* seem to be able to elicit beneficial modifications of skeletal muscular mass, architecture, and quality in active elderly subjects in connection with an amelioration of the functional performances (strength, power, neuromuscular activation, etc.). In particular, a significant reduction of *IMAT* was evident after the two training interventions, a fact that led to the amplification of the percent changes of *ACSA* of the muscles when they were calculated without considering *IMAT*.

*IMAT* is an important predictor of muscle metabolism and also appears to be a modifiable muscle risk factor: adipose tissue stored in ectopic locations, as in the muscle, is connected with impaired glucose tolerance, chronic inflammation, and increased total cholesterol (Prior et al., 2007; Durheim et al., 2008; Koster et al., 2010). Physical activity and resistance or endurance training appear to be effective countermeasures against increases in *IMAT*. The exercise protocol proposed in our study has positively influenced *IMAT*: we can therefore speculate that the exercise carried out at high intensity reduces modifiable muscle risk factors.

The two training modalities caused a homogeneous increase of *ACSA* of the quadriceps at different percentages of the total muscle length. By the same token, the percent increase of *Vol* of the different bellies of the quadriceps turned out to be homogeneous. θ*_p_* underwent an expected increase with both training modalities with a consensual increase of *PCSA* in *IRT*. However, specific strength per unit of *PCSA* did not change and the observed increases of strength seemed to be attributed to a parallel improvement of neuromuscular activation and muscle hypertrophy only after *IRT*.

We can therefore consider that both *HIT* and, especially, *IRT* induce beneficial modification on different systems with the final effect to counteract most of the causes of the morphological and functional consequences of sarcopenia.

## Data Availability Statement

The raw data supporting the conclusions of this manuscript will be made available by the authors, without undue reservation, to any qualified researcher.

## Ethics Statement

The protocol and the methods of the study were approved by the Regional Review Board (approval on June 18, 2013) and written informed consent was obtained from each subject before entering the study.

## Author Contributions

PB, ET, and CC conceived the study. PB, ET, and EC collected the data. PB, ET, EC, and AR analyzed the data. PB and CC wrote the first draft of the manuscript. All authors approved final version of the manuscript.

## Conflict of Interest

The authors declare that the research was conducted in the absence of any commercial or financial relationships that could be construed as a potential conflict of interest.

## Abbreviations

*%Act*: percent neuromuscular activation
θ^*p*^: pennation angle of muscle fibers
*ACSA*: anatomical cross-sectional area
*BMI*: body mass index
*HIT*: high-intensity interval training
*IMAT*: intermuscular adipose tissue
*IRT*: isoinertial resistance training
*L*_*f*_: fascicle length
*MVC*: maximal voluntary contraction
$\dot{V}$O_2_: oxygen uptake
$\dot{V}$O_2max_: maximal oxygen uptake
*PCSA*: physiological cross-sectional area
*QF*: *quadriceps femoris*
*RF*: *rectus femoris*
*RT*: resistance training
*T*_C_, *T*_*MVC*_: Maximal concentric and isometric muscular torques
*T*_*m*_: muscle thickness
*T*_*MVCcor*__r_: corrected maximal isometric force exerted on the patellar tendon calculated from *T*_*MVC*_ at 90° of knee flection
*VI*: *vastus intermedius*
*VL*: *vastus lateralis*
*VM*: *vastus medialis*
*Vol*: muscle volume
