# Supplementary material for: Effects of High-Intensity Interval Training and Isoinertial Training on Leg Extensors Muscle Function, Structure, and Intermuscular Adipose Tissue in Older Adults
Source: Front Physiol. 2019 Oct 9;10:1260. doi: 10.3389/fphys.2019.01260 (PMC6794371; doi:10.3389/fphys.2019.01260)
Supplement: Supplementary file 1 [file Table_1.DOCX]

|  | *pre HIT* | *post HIT* | *pre IRT* | *post IRT* |
| --- | --- | --- | --- | --- |
| ACSA at 25% LF (cm^2^) | 42.9 ± 7.6 | 46 ± 7.9* | 44.8 ± 7.7 | 47.9 ± 8.8* |
| ACSA at 50% LF (cm^2^) | 59.8 ± 10.8 | 62.1 ± 10.7* | 58.6 ± 9.9 | 61.7 ± 9.4* |
| ACSA at 75% LF (cm^2^) | 46.3 ± 10.5 | 48.9 ± 9.4* | 49 ± 9 | 52.4 ± 9.8* |
| Vol (cm^3^) | 804.6 ± 202.7 | 847 ± 204* | 781.8 ± 193.7 | 850.2 ± 187.5* |
| VM (cm^3^) | 187.8 ± 31.4 | 191.1 ± 32.9 | 195.1 ± 43.4 | 214 ± 39.7 |
| VI (cm^3^) | 266.4 ± 85.5 | 283.3 ± 85.1 | 249.9 ± 68 | 273 ± 70.7 |
| VL (cm^3^) | 272.7 ± 71.4 | 290.6 ± 76.2 | 261.6 ± 75.3 | 282.5 ± 73.5 |
| RF (cm^3^) | 77.7 ± 30.6 | 82.1 ± 29.9 | 75.2 ± 27 | 80.8 ± 24.3 |
| IMAT at 50% LF (cm^2^) | 5.4 ± 2.6 | 3.7 ± 1.7* | 3.1 ± 1.7† | 1.7 ± 1.2*‡ |
| SCAT at 50% LF (cm^2^) | 69.1 ± 17.8 | 62.2 ± 17.7* | 62.8 ± 17.6 | 62 ± 17.4 |
| ACSA at 50% LF NO IMAT (cm^2^) | 54.4 ± 8.2 | 58.3 ± 8.9* | 55.5 ± 8.2 | 60 ± 8.2* |
| T*_MVC_* ISO 90° (Nm) | 169 ± 34.1 | 164.8 ± 30.6 | 165.9 ± 37.5 | 177.4 ± 42.4* |
| T*_C_* 120 ° ^sec-1^ (Nm) | 130.5 ± 22.5 | 133.5 ± 23.9 | 131.2 ± 23.1 | 139.3 ± 22.7*‡ |
| *θ_p_* VL (deg) | 10.4 ± 1.1 | 11.9 ± 1.6* | 11.2 ± 1.2 | 13.1 ± 1.7* |
| PCSA at 50% LF (cm^2^) | 77.3 ± 23.2 | 77.8 ± 24.3 | 69.6 ± 16.7 | 78.3 ± 19.1* |
| Strength//ACSA – 90° (N/cm^2^) | 60.3 ± 6.1 | 57.1 ± 7* | 60.4 ± 5.3 | 61.3 ± 9.4 |
| Strength/PCSA – 90° (N/cm^2^) | 66.4 ± 6.1 | 60.8 ± 7.5* | 63.8 ± 5.6 | 63 ± 9 |
| Leg Activation (%) | 76.2 ± 16.5 | 86.5 ± 11.1 | 82 ± 10.6 | 92.2 ± 5.3* |
| Arm Activation (%) | 55.2 ± 10 | 55.6 ± 11.9 | 50.6 ± 13.7 | 51.4 ± 24 |

**Table 1**. Average values before (*pre HIT*) and after (*post HIT*) high intensity interval training and before (*pre IRT*) and after (*post IRT*) isoinertial resistance training (mean, SD; n=12).

ACSA at xx% LF: anatomical cross-sectional area at 75 %, 50 % and 25 % of the femur length (LF). Vol: Muscle volume of total quadriceps femoris; VM: vastus medialis volume; VI: vastus intermedius volume; VL: vastus lateralis volume; RF: rectus femoris volume; IMAT at 50% LF: intermuscular adipose tissue at 50 % of femur length; SCAT at 50% LF: subcutaneous adipose tissue at 50 % of femur length; ACSA at 50% LF NO IMAT: ACSA without IMAT; T*_MVC_* ISO 90°: Maximal isometric muscular torque; T*_C_* 120 ° ^sec-1^ : Maximal concentric muscular torque at 120 ° sec^-1^ speed of knee extension; θp : Pennation angle of VL muscle fibers; PCSA at 50% LF: physiological cross-sectional area calculated at 50 % of femur length; Strength/ACSA – 90°: specific strength, ratio between torque and ACSA; Strength/PCSA – 90°: torque per unit of PCSA; Leg Activation: quadriceps femoris per-cent neuromuscular activation; Arm Activation: biceps brachialis per-cent neuromuscular activation. *: Pre - Post significantly different values (P< 0.05); †: Significantly different values between Pre conditions (P< 0.05); ‡: Significantly different values between Post conditions (P< 0.05).
